# Cancer-associated fibroblast exosomes promote chemoresistance to cisplatin in hepatocellular carcinoma through circZFR targeting signal transducers and activators of transcription (STAT3)/ nuclear factor -kappa B (NF-κB) pathway

**DOI:** 10.1080/21655979.2022.2032972

**Published:** 2022-02-09

**Authors:** Yun Zhou, Weiwei Tang, Han Zhuo, Deming Zhu, Dawei Rong, Jin Sun, Jinhua Song

**Affiliations:** a Department of Ultrasonography, Nanjing First Hospital, Nanjing Medical University, Nanjing, China; b Key Laboratory of Liver Transplantation, Chinese Academy of Medical Sciences; Nhc Key Laboratory of Living Donor Liver Transplantation, Hepatobiliary Center, the First Affiliated Hospital of Nanjing Medical University, Nanjing, China; c Department of Nuclear Medicine, The First Affiliated Hospital of Nanjing Medical University, Nanjing, China

**Keywords:** Cancer-associated fibroblast exosomes, circZFR, hepatocellular carcinoma, cisplatin resistance

## Abstract

Chemoresistance in hepatocellular carcinoma (HCC) has been found to be influenced by exosomal transport of circRNAs. However, the role of circZFR in HCC chemoresistance still remains unclear. In the present study, circZFR was highly expressed in cisplatin (DDP)-resistant HCC cell lines and could regulate DDP resistance of the HCC cells. Also, circZFR was highly expressed in cancer-associated fibroblast (CAFs) and the exosome of CAFs. In addition, supplementation of CAFs in culture medium could promote DDP resistance of HCC cells. In vivo tumor xenograft experiments showed that knockdown of circZFR inhibited tumor growth and weakened DDP resistance, while CAFs-derived exosomes incubation increased the expression of circZFR, inhibited the STAT3/NF-κB pathway, promoted tumor growth, and enhanced DDP resistance. In general, CAFs-derived exosomes deliver circZFR to HCC cells, inhibit the STAT3/NF-κB pathway, and promote HCC development and chemoresistance. The results provided a new sight for the prevention and treatment of chemoresistance in HCC.

## Graphical abstract

**Figure:**
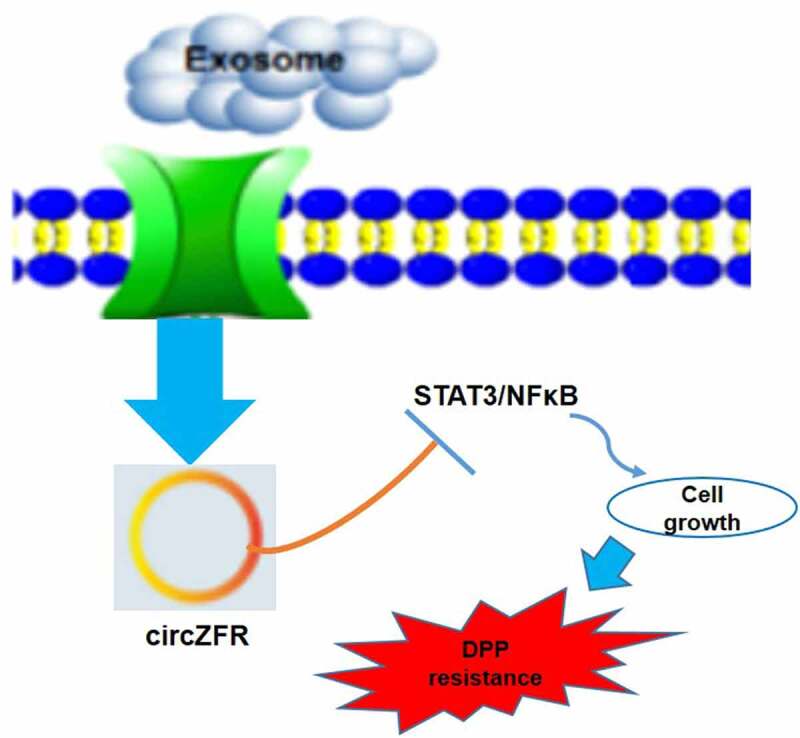

## 1. Introduction

Hepatocellular carcinoma (HCC) is the sixth most common cancer worldwide and the fourth most common cause of cancer-related death [1], and its incidence is on the rise worldwide. Although multiple therapeutic strategies including bioactive compounds isolated from fungi have greatly improved the survival rate of patients with HCC [2], the invasion and metastasis of tumors and the resistance to conventional chemotherapeutic agents (such as cisplatin) remain obstacles to HCC treatment [3]. DDP is the first-line chemotherapy drug in the current clinical treatment of HCC, and resistance to DDP seriously affects the efficacy of chemotherapy treatment, which is also one of the main reasons that the 5-year survival rate of patients with HCC is less than 20% [4]. However, the mechanism of HCC resistance to DDP is not clear at present, so it is urgent to screen the biomarkers and targets of DDP resistance to improve the efficacy of HCC chemotherapy.

Circular RNAs (circRNAs) are a class of endogenous non-coding RNAs (ncRNAs) that exhibit complex tissue-specific and stage-specific expression in eukaryotic transcriptome. So far, numerous studies have shown that circRNAs play an important role in tumor formation, progression, recurrence, and drug resistance [5,6]. In HCC, there has been a lot of coverage. For example, circ_0008274 [7], circ_0008583 [8], circMTO1 [9], circRNA-5692 [10], and circ_0001955 [11] have been reported to influence HCC progression. Various circRNAs have also been reported to mediate chemoresistance of HCC, such as circRNA_102272 [12], circRNA_101505 [13], and circRNA-SORE [14]. All these studies showed circRNAs could be promising therapeutic target for chemoresistance.

CircZFR has been proved to have a close relationship with HCC and promoted cell proliferation and migration of HCC by regulating miR3619-5p/CTNNB1 axis or miR-511/AKT1 axis, thus promoting the progression of HCC [15,16]. It has been reported that the depletion of circZFR can contribute DDP inhibition of non-small-cell lung cancer (NSCLC) tumors [17], but whether it is involved in DDP resistance in HCC has not been reported. As an important part of tumor microenvironment, cancer-associated fibroblasts (CAFs) interact with cancer cells to promote tumorigenesis and development, and are considered as potential targets for anticancer therapies. Studies have confirmed that CAFs can promote the development of HCC through its exosomes [18,19]. CAFs-derived exosomes play a crucial role in the communication between CAFs and cancer cells and other cells of the TME [20]. In addition, chemoresistance of CAFs-derived exosomes has been demonstrated in a variety of cancers [21–24]. How CAFs-derived exosomes promote HCC progression through circRNAs is the focus of this study.

In the present study, we aim to explore the effect and mechanism of exosomal circZFR from CAFs- on chemoresistance to HCC. As circZFR was highly expressed in the exosomes of cisplatin (DDP)-resistant HCC cell lines. We hypothesized that CAFs-derived exosomes deliver circZFR to HCC cells and promote HCC development and chemoresistance. Subsequently, a series of molecular and cellular experiments were performed to test the function of circZFR. Our study will provide a new insight for the prevention and treatment of chemoresistance in the therapy of HCC.

## 2. Materials and methods

### 2.1 Cell culture

Human normal hepatocyte cell line L02, HCC cell lines Huh7 and MHCC97L were obtained from the Chinese Academy of Sciences Cell Bank (China), as well as the fibroblast cell lines. The cells were cultured in Dulbecco’s modified Eagle’s medium (DMEM, Gibco, USA), containing 10% fetal bovine serum, penicillin (100 U/mL), and streptomycin (100 mg/mL) at 37°C with 5% CO_2_. Huh7 or MHCC97L cells in logarithmic phase were inoculated into 6-well plates. After cells were adhered overnight, 1 μg/mL cisplatin (DDP, Sigma, USA) was added. Then, the drug concentration was increased by 0.5 μg/mL step after cell growth stably. After about 4 months of induction, the cells could grow and generate stably in RPMI1640 medium containing 4 μmol/L DDP. DDP-resistant cell lines were named Huh7/DDP and MHCC97L/DDP.

### 2.2 Real-time -PCR (RT-PCR)

The total RNA of cells was extracted with Trizol (Takara, Japan). RNA reverse transcription kit (Takara) was used to reverse transcribe cDNA, and 1 µg cDNA and SYBR Green RT-PCR kit (Takara) were taken for RT-PCR. The reaction conditions were pre-denaturation at 95°C for 3 min, 40 cycles (95°C 30s, 58°C 45s), and extended at 72°C for 6 min [25]. Following primers synthesized by Shanghai Sangon Biotech Co., Ltd. (China) were used: circZFR forward 5’-TGCCACCATTTATCCAACTG-3’ and reverse 5’-CCACTCGCAAAACTCCTTTC-3’; GAPDH (internal control) forward, 5’-CCAGGTGGTCTCCTCTGA-3’ and reverse 5’-GCTGTAGCCAAATCGTTGT-3’. The 2^−ΔΔCt^ method was used to calculate the relative expression of targets. Repeat at least three times for each sample.

### 2.3 RNA transfection [26]

Corresponding lentiviruses expressing sequence shRNAs specific to circZFR (sh-circZFR) was designed and synthesized by Shanghai Integrated Biotech Solutions Co., Ltd. (China) to knockdown circZFR in Huh7 and MHCC97L cell lines. Nontarget shRNA lentiviruses (sh-NC) were used as the negative control. Plasmid overexpression of circZFR was obtained from GeneChem Company (China). Transfections were conducted by the use of Lipofectamine 2000 (Invitrogen, USA) based on the manufacturer’s protocol. Lentiviruses-transfected cells were selected by puromycin (1:1000, Solarbio, China) at least 48 h and thereafter cultured normally.

### 2.4 Cell counting Kit −8 (CCK-8) assay [27]

The CCK-8 kit was purchased from Boster Biological Technology Co., Ltd. (China) and operated according to the supplier’s instructions. Cells in logarithmic growth phase were seeded into 96-well plates. 1 μg/ml, 2 μg/ml, 4 μg/ml, 8 μg/ml, and 16 μg/ml DDP were added after cell attachment. After 48 h of culture, the optical concentration (OD) values at 450 nm were measured with a microplate analyzer.

### 2.5 Apoptosis assay [28]

After transfection, cells of each group were collected, prepared into single-cell suspension, and 2 μg/ mL DDP was added for 48 h. Annexin V-fluorescein isothiocyanate (FITC)/propidium iodide (PI) (BD Pharmingen, USA) were added into the cell suspension according to the instructions, and placed in dark for 15 min. Cell apoptosis was detected by flow cytometry in each group, and early apoptotic cells, late apoptotic cells, and dead cells were counted. Meanwhile, there were three replicates of each experiment.

### 2.6 Exosome collection and detection [29]

CAFs and normal fibroblasts (NFs) were cultured in exosome-free media in the initial. Media from cells and phosphate buffer solution (PBS) washed cells was collected and together centrifuged for 5 min, and then supernatant was centrifuged for 20 min. Exosomes were isolated with the ExoQuick-TC™ system (System Bioscience, USA) according to the protocol.

Exosomal protein was measured via a BCA assay, and 10 μg exosomes were placed on copper grid. Exosomes were wicked off to create a thin layer prior to addition of a thin layer of 2% uranyl acetate in water. Grids were allowed to dry overnight, and TEM performed the next day. Exosomes were resuspended in PBS and particle sizes were measured using the Beckman Coulture® Delsa™ Nano S Particle Analyzer. The identified exosomes were co-cultured in HCC cell medium with 20 µM DMSO or GW4869.

### 2.7 Western blot assay [30]

Cells were collected and disrupted with RIPA cleavage buffer (Thermo Fisher Scientific, USA). The lysates were collected after centrifugation and the protein concentration was quantified with BCA kit (Takara). 50 mg of proteins were added to sodium dodecyl sulfate polyacrylamide gel electrophoresis (SDS-PAGE) and then were transferred onto polyvinylidene difluoride (PVDF) membranes (Millipore, USA). These membranes were blocked in 5% nonfat milk for 1 h at room temperature and then were treated as antibody protocol described overnight at 4°C. The following antibodies purchased from Abcam and Cell Signal (USA) were used: anti-CD9, anti-CD63, anti-CD81, anti-STAT3, anti-phosphor-STAT3, anti-NF-κB, anti-phosphor-NF-κB, and anti-GAPDH. Moreover, respective secondary antibody was used to incubate these membranes according to protocol. The protein bands were quantified with ECL system (Thermo Fisher Scientific) and were analyzed by Image J software.

### 2.8 Immunostaining [31]

CAFs-derived exosomes were labeled with a fluorescent dye CM-Dil and incubated with HCC cells for 12 h at 37°C. Tumor tissue was embedded in paraffin and sliced into 7 µm slices. Cells or tissues were fixed with methanol and permeabilized with 0.1% Triton X-100 in PBS for 20 min. The tissues were incubated with TUNEL, Ki67 and anti-cl-Caspase-3 primary antibody overnight at 4°C, and then incubated in fluorochrome-conjugated or normal secondary antibodies for 2 h at room temperature. DAPI was used to counterstain the cell nuclei. Images were captured with a Laser confocal microscope (Olympus, Japan) in five different fields for each sample. After incubation with secondary antibody of cleaved Caspase-3, color was developed with DAB color development kit (Boster), and pictures were taken with ordinary light microscope.

### 2.9 Tumor xenograft assay [32]

All animal procedures were approved by the Ethics Committee of the First Affiliated Hospital of Nanjing Medical University. A total of 36 nude mice (4 weeks old) were intrathoracically injected 100 μL of PBS with 1 × 10^5^ transfected Huh7 cells (n = 6). The mice were killed after 5 weeks. The tumor tissues were weighed and calculated the volume. Tumor tissues were also processed and sectioned for pathological assessment.

### 2.10 Statistical analysis

All data are presented as the means ± standard deviation (SD). The data statistical significance analyses and statistical mapping were performed using Prism GraphPad 8.0 software. Data were analyzed using one-way ANOVA and Student’s t-test. P < 0.05 was considered to be statistically significant.

## 3. Results

This study aims to explore the effect and mechanism of exosomal circZFR from CAFs- on chemoresistance to HCC. We found that circZFR was highly expressed in DDP-resistant HCC cell lines, and circZFR could affect the DDP resistance of HCC cells. CircZFR was highly expressed in cancer-associated fibroblast (CAFs), and the supplementation of CAFs in culture medium could promote DDP resistance of HCC cells. In vivo tumor xenograft experiments showed that knockdown of circZFR inhibited tumor growth and weakened DDP resistance, while CAFs-derived exosomes increased circZFR level, inhibited the STAT3/NF-κB pathway, promoted tumor growth and enhanced DDP resistance. In a word, CAFs-derived exosomes deliver circZFR to HCC cells, inhibit the STAT3/NF-κB pathway, and promote HCC development and chemoresistance.

### 3.1 CircZFR was highly expressed in DDP-resistant HCC cells

**Figure 1.**
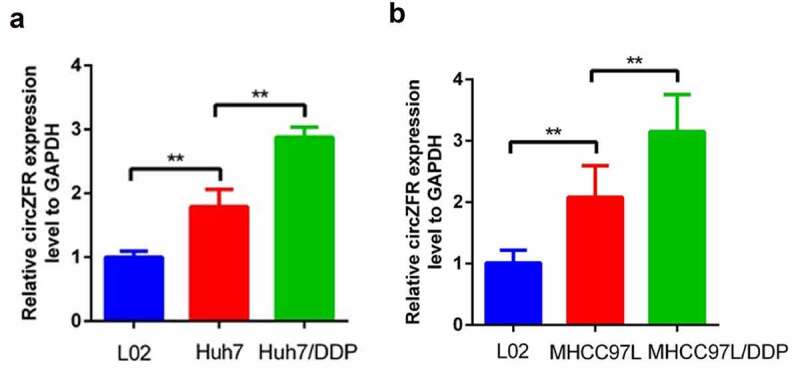
**The expression of circRNA in normal liver cells, HCC cells and DDP resistant HCC cells**. (a) HCC cell lines Huh7; (b) HCC cell lines MHCC97L, **P < 0.01.

In order to determine whether circZFR is involved in the DDP resistance of HCC cells, RT-qPCR was used to detect the expression of circZFR in normal liver cells L02, HCC cells (Huh7 and MHCC97L) and DDP-resistant HCC cells. The expression of circZFR was shown in Figure 1. The results showed that circZFR in both HCC cell lines was significantly higher than that in L02 cell (*P* < 0.01). Compared with HCC cells, circZFR expression levels were significantly increased in both DDP resistant HCC cells (*P* < 0.01). This indicated that circZFR may not only be involved in the occurrence of HCC, but also in the DDP resistance of HCC.

### 3.2 CircZFR promoted DDP resistance in HCC cells

**Figure 2.**
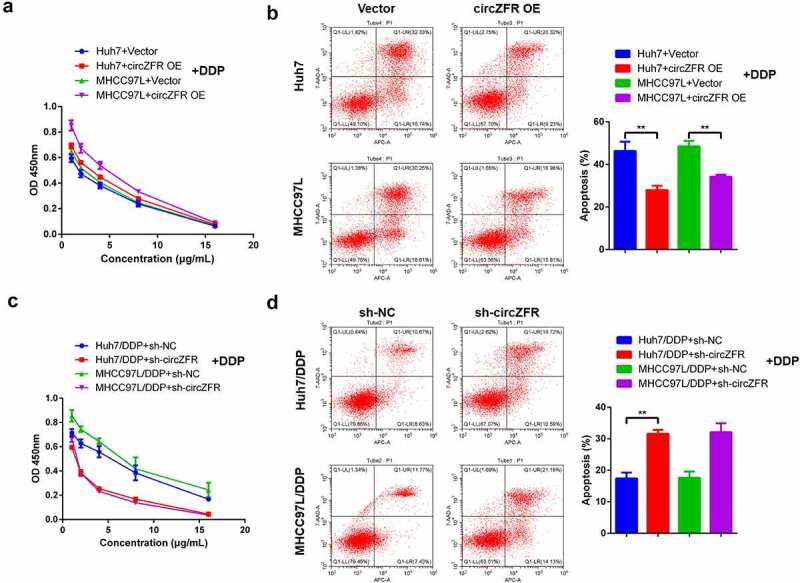
**CircZFR promoted DDP resistance in HCC cells**. Cell viability (a) and apoptosis (b) of circZFR overexpressed or empty vector transfected HCC cells treated with different concentrations of DDP for 48 h; Cell viability (c) and apoptosis (d) of sh-circZFR or sh-NC transfected DDP resistant HCC cells treated with different concentrations of DDP for 48 h; **P < 0.01.

After the elevated expression level of circZFR was confirmed in DDP resistant HCC cells, circZFR overexpression vector was transfected in HCC cells. ALso, circZFR shRNA vector was transfected in DDP resistant HCC cells to explore the influence of circZFR on DDP resistance of HCC cells. After transfected cells were treated with DDP at different concentrations for 48 h, the proliferation and apoptosis of cells in each group were shown in Figure 2. The cell viability of circZFR overexpressed HCC cells after DDP treatment was higher than that of empty vector transfected HCC cells (Figure 2a), and the apoptosis rate was significantly reduced (*P* < 0.01, Figure 2b). Compared with sh-NC transfected HCC cells, sh-circZFR transfected HCC cells showed significantly decreased cell viability (Figure 2c) and increased apoptosis rate (*P* < 0.01, Figure 2d). The results were the same in Huh7 and MHCC97L cells, mutually verifying the credibility of circZFR promoting DDP resistance in HCC cells.

### 3.3 CAFs-derived exosomes might regulate circZFR DDP resistance

**Figure 3.**
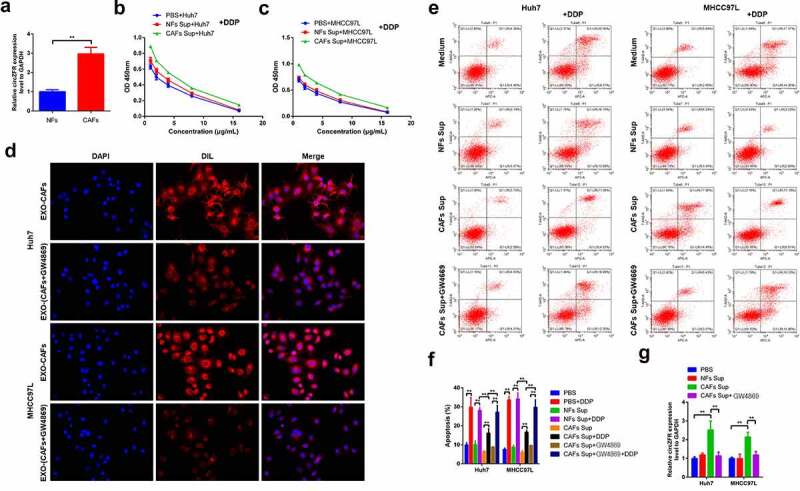
**CAFs-derived exosomes might be involved in the regulation of circZFR expression and DDP resistance in HCC cells**. (a) The expression of circZFR in CAFs was significantly higher than that in NFs; CAFs significantly increased the cell viability of Huh7 (b) and MHCC97L (c) cells after DDP treatment; (d) CM-DIL labeled exosomes in HCC cells were significantly reduced after GW4869 treatment; (e) and (f) Blocking the secretion of CAFs exosomes significantly increased the apoptosis rate of HCC cells cultured by medium supplemented with CAFs; (g) The blocking of CAFs exosomes secretion significantly decreased the expression level of circZFR in HCC cells cultured by medium supplemented with CAFs; **P < 0.01.

In order to discuss the possible mechanism of circZFR involvement in DDP resistance of HCC cells, CAFs and NFs were isolated. CircZFR was found to be expressed in both fibroblasts, and the expression of circZFR in CAFs was significantly higher than that in NFs (*P* < 0.01, Figure 3a). After 4 days’ culture of HCC cells with control medium or medium containing NFs or CAFs, the cells were treated with different concentrations of DDP for 48 h to detect the viability of HCC cells. In both Huh7 and MHCC97L cells, the viability of cells cultured by medium supplemented with CAFs was significantly higher than that cultured by medium supplemented with NFs and control medium (Figure 3b and c). CircZFR was highly expressed in CAFs, and CAFs could promote DDP resistance of HCC cells.

In order to determine whether CAFs-derived exosomes affected the DDP resistance of HCC cells, GW4869 was added to the medium supplemented with CAFs to block CAFs exosomes secretion. CM-DIL (red) was used to label exosomes. Immunofluorescence results showed that GW4869 treatment significantly reduced the fluorescence of CM-DIL in HCC cells (Figure 3d), indicating that GW4869 treatment could block the secretion of exosomes by CAFs. After the same treatment with DDP for 48 h, cell apoptosis was detected in each group, and the results were shown in Figure 3e and **f**. DDP treatment significantly increased the apoptosis rate of cells in each group (*P* < 0.01). Compared with the medium supplemented with NFs, the apoptosis rate of HCC cells cultured in the medium supplemented with CAFs significantly decreased after DDP treatment (*P* < 0.01). It was worth noting that after using GW4869to block the secretion of exosomes of CAFs, the apoptosis rate of the two kinds of HCC cells treated with DDP was significantly increased compared with that cultured with medium supplemented with CAFs alone (*P* < 0.01). CircZFR was detected in each group again, and the expression of circZFR was significantly increased in CAFs cultured cells (*P* < 0.01) and decreased after the secretion of CAFs exosomes were blocked in both Huh7 and MHCC97L cells (*P* < 0.01, Figure 3g). These results indicated that CAFs-derived exosomes can regulate the level of circZFR in HCC cells and affect their DDP resistance.

### 3.4 CircZFR inhibited the STAT3/NF-κB pathway and promoted DDP resistance of HCC cells

**Figure 4.**
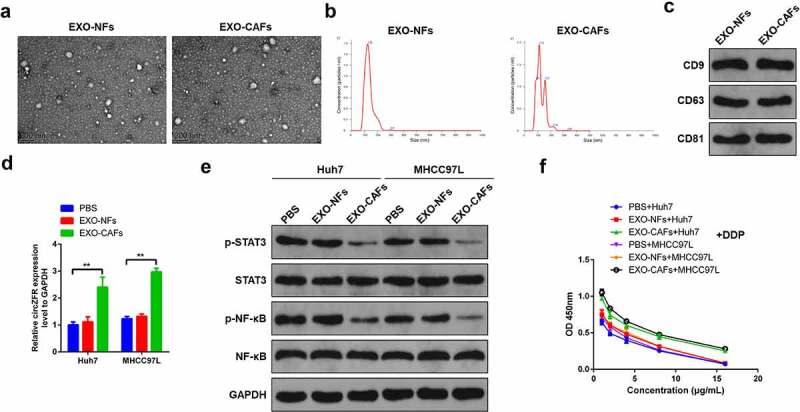
**CAFs-derived exosomes deliver circZFR to HCC cells and inhibit the STAT3/NF-κB pathway, promoting DDP resistance of HCC cells**. (a) NFs and CAFs-derived exosomes photographed by transmission electron microscopy; (b) The diameter distribution of exosomes ranged around 120 nm; (c) The expressions of CD9, CD63 and CD81 exosome markers in the isolated exosomes were detected by Western blot; (d) CAFs-derived exosomes significantly increased the expression level of circZFR in HCC cells; (e) The phosphorylation levels of STAT3 and NF-κB in HCC cells were significantly decreased by CAFs-derived exosomes culture; (f) CAFs-derived exosomes culture could significantly improve the viability of HCC cells after DDP treatment; **P < 0.01.

To further determine and explore the influence mechanism of CAFs exosomes on DDP resistance of HCC cells, exosomes derived from NFs and CAFs were isolated and purified. Isolated exosomes were examined for size and structure via transmission electron microscopy (Figure 4a). The diameter distribution of exosomes was analyzed by nanoparticle tracking analysis, and particles ranged around 120 nm in diameter (Figure 4b). Subsequently, the expressions of CD9, CD63, and CD81 exosome markers in the isolated exosomes were detected by Western blot (Figure 4c). Based on these, it was concluded that exosomes of CAFs and NFs were successfully isolated and purified. The expression of circRNA in HCC cells was detected after 4 days’ culture in which exosomes of CAFs or NFs was used. It was found that CAFs-derived exosomes significantly increased the expression level of circZFR in HCC cells (*P* < 0.01, Figure 4d). Then, protein phosphorylation levels of STAT3 and NF-κB in each group of cells were performed, and the results showed that the phosphorylation levels of STAT3 and NF-κB in HCC cells were significantly decreased by CAFs-derived exosomes culture (*P* < 0.01, Figure 4e). The viability of cells in each group was also detected and found that CAFs-derived exosomes culture could significantly improve the viability of HCC cells after DDP treatment (Figure 4f), suggesting that CAFs-derived exosomes promote DDP resistance of HCC cells.

### 3.5 *CAFs-derived exosomes promote DDP resistance through circZFR* in vivo

**Figure 5.**
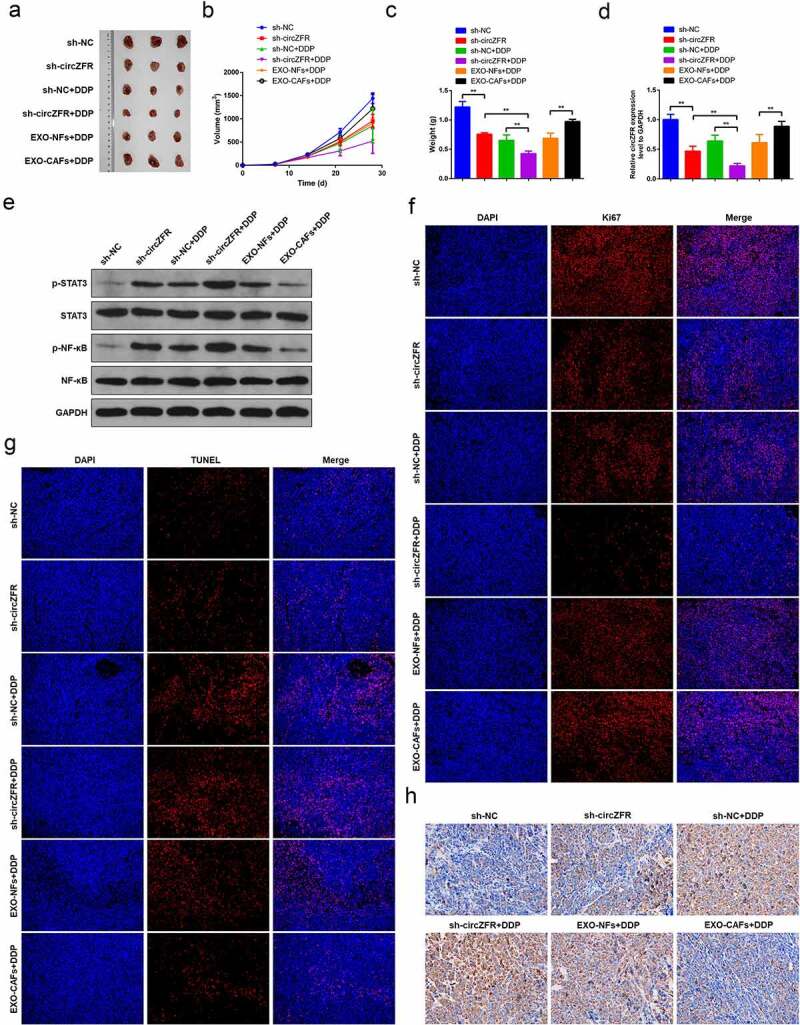
**In vivo tumor transplantation confirmed that CAFs-derived exosomes promote DDP resistance of HCC through circZFR**. (a) Tumor tissue photos of each group; Tumor tissue volumes (b) and weights (c) in each group; (d) The expression level of circZFR in tumor tissues of each group; (e) Phosphorylation levels of STAT3 and NF-κB in tumor tissues of each group; Ki67 (f) and TUNEL (g) immunofluorescence staining of tumor tissues in each group; H: The levels of cleaved Caspase-3 in tumor tissues of each group detected by immunohistochemistry, **P < 0.01.

Sh-circZFR lentivirus was constructed and transfected in Huh7 cells to knockdown the expression level of circZFR. Sh-circZFR or sh-NC transfected Huh7 cells and Huh7 cells cultured with CAFs-derived or NFs-derived exosomes were used for in vivo tumor transplantation experiments. Part of animals were treated with DDP to observe the drug resistance of Huh7 cells. The animals were sacrificed 35 days after the tumor transplantation, and the tumor was removed and photographed (Figure 5a). Tumor volumes (Figure 5b) and weights (Figure 5c) of each group were shown. Knockdown of circZFR could significantly reduce tumor volume and weight and enhance the therapeutic effect of DDP, while CAFs-derived exosomes resist DDP treatment and increase tumor volume and weight.

After that, the expression level of circZFR was performed in tumor tissues of each group, as shown in Figure 5d. It was confirmed that sh-circZFR lentivirus successfully knocked down the expression of circZFR in tumor tissues, and CAFs-derived exosomes increased the level of circZFR. The phosphorylation level of STAT3 and NF-κB in tumor tissues increased with the decrease of circRNA level (Figure 5e). Proliferation and apoptosis of tumor tissues were also examined by immunostaining. The level of Ki67 represented the state of tumor proliferation, and tumor proliferation was inhibited with the decrease of the expression level of circZFR (Figure 5f). Meanwhile, with the decrease of circZFR level, tumor apoptosis and the expression of cleaved Caspase-3 were significantly increased (Figure 5g and h). To sum up, circZFR knockdown and DDP treatment significantly inhibited tumor proliferation and promoted tumor apoptosis, and circZFR knockdown could enhance the efficacy of DDP. However, CAFs-derived exosomes increased the level of circZFR, promoted tumor proliferation and inhibited apoptosis, and enhanced tumor DDP resistance.

## 4. Discussion

HCC is one of the most common primary tumors, and its high metastatic rate and high recurrence rate cause a treatment bottleneck [33]. Chemotherapy failure due to drug resistance is an important factor, which leads to low survival rate in patients with HCC [34]. From the perspective of precision medicine, there is an urgent need to find new molecular targets or signaling pathways to reduce the chemoresistance of HCC patients and improve the therapeutic effect.

CAFs and their exosomes have been shown to be involved in tumor progression and chemoresistance in a variety of malignancy [22,35,36]. For example, Yang et al. reported that exosomal circEIF3K from cancer-associated fibroblast promotes colorectal cancer (CRC) progression via miR-214/PD-L1 axis [37]. In addition, through secreting various growth factors and cytokines, CAFs contribute to the ECM remodeling, stem features, angiogenesis, immunosuppression, and vasculogenic mimicry (VM), which reinforce the initiation and development of HCC [38]. However, the role and mechanism of circRNA from CAFs-derived exosomes in HCC chemoresistance are rarely reported.

CircZFR has been shown to promote tumor progression in a variety of malignancies [39–43], but the mechanism of its involvement in drug resistance in HCC and its relationship with CAFs-derived exosomes remain unknown. Our results confirmed the hypothesis that CAFs-derived exosomes increased the expression level of circZFR in HCC cells and promoted DDP resistance of HCC cells. CircZFR was highly expressed in CAFs-derived exosomes and transported to HCC cells to suppress the STAT3/ NF-κB pathway and promote DDP resistance.

In this study, circZFR was highly expressed in DDP resistant HCC cells, and overexpression of circZFR could enhance DDP resistance of HCC cells, while knockdown of circZFR could inhibit DDP resistance of HCC cells. CircZFR is a newly discovered circular RNA, and its carcinogenic effect has been demonstrated in a variety of malignant tumors [40,44]. In HCC, circZFR can target miRNA and proteins to promote HCC cell proliferation and migration, leading to cancer development [15,16]. The study found that circZFR can affect the efficacy of DDP in NSCLC [17], but there is currently no study on the mechanism of circZFR affecting chemoresistance in HCC. Our results confirmed that circZFR is involved in the chemoresistance of HCC and may play a role through inhibition of the STAT3/NF-κB pathway. STAT3 and NF-κB pathways play an important role in the development and progression of cancer, and are closely related to proliferation, metastasis, and autophagy of HCC [45–47]. Due to the short time of discovery of circZFR, its interaction with STAT3 and NF-κB pathways has not been reported. However, whether circZFR directly targets STAT3 and NF-κB, as well as its potential upstream and downstream molecules, remains to be further explored.

Cellular interactions between cancer cells and surrounding stromal cells in the tumor microenvironment (TME) play an important role in regulating tumor progression and therapeutic response [48,49]. As an important component of TME, CAFs interact with cancer cells and promote tumor genesis and development. The chemoresistance role of CAFs in HCC has been confirmed by a large number of data [50–52]. Our study found that the elevated expression of circZFR in CAFs can promote DDP resistance of HCC cells. The carcinogenic effect of CAFs-derived exosomes in promoting HCC proliferation and metastasis has been confirmed [18,19,53], and our results confirmed their role in promoting chemoresistance in HCC. In addition, single-cell RNA sequencing (scRNA-seq) analyses provides critical information on cell identity and function [54]. In the further study, single-cell transcriptomic strategy will be used to study the function of circZFR in the subcells of HCC tissues.

## 5. Conclusion

In conclusion, this study indicated that circZFR can promote DDP resistance in HCC cells. In addition, CAFs-derived exosomes promote chemoresistance by increasing the expression of circZFR in HCC cells through STAT3/NF-κB pathway. The findings of this study provided new molecular targets for enhancing the response to chemotherapy in HCC, and provided new possibilities for improving the therapeutic effect and survival rate of HCC.

## Supplementary Material

Supplemental Material

## Data Availability

The datasets analyzed during the current study are available from the corresponding author on reasonable request.

## Highlights

CircZFR was highly expressed in DDP-resistant HCC cells and promoted DDP resistance.

CAFs-derived exosomes promote DDP resistance of HCC through circZFR *in vitro* and *in vivo*.

CAFs-derived exosomes deliver circZFR to HCC cells and inhibit the STAT3/NF-κB pathway.
