# Supplementary figures and images for: Cancer-associated fibroblast exosomes promote chemoresistance to cisplatin in hepatocellular carcinoma through circZFR targeting signal transducers and activators of transcription (STAT3)/ nuclear factor -kappa B (NF-κB) pathway
Source: Bioengineered. 2022 Feb 9;13(3):4786–97. doi: 10.1080/21655979.2022.2032972 (PMC8973934; doi:10.1080/21655979.2022.2032972)

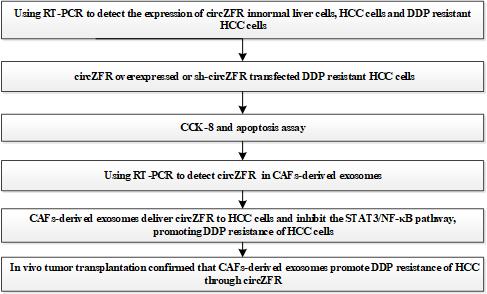

Supplement: Supplemental Material [file KBIE_A_2032972_SM5618.jpg]
